# Acetylcholine-gated current translates wake neuronal firing rate information into a spike timing-based code in Non-REM sleep, stabilizing neural network dynamics during memory consolidation

**DOI:** 10.1371/journal.pcbi.1009424

**Published:** 2021-09-20

**Authors:** Quinton M. Skilling, Bolaji Eniwaye, Brittany C. Clawson, James Shaver, Nicolette Ognjanovski, Sara J. Aton, Michal Zochowski

**Affiliations:** 1 Biophysics Program, University of Michigan, Ann Arbor, Michigan, United States of America; 2 Applied Physics Program, University of Michigan, Ann Arbor, Michigan, United States of America; 3 Department of Molecular, Cellular, and Developmental Biology, University of Michigan, Ann Arbor, Michigan, United States of America; 4 Department of Physics, University of Michigan, Ann Arbor, Michigan, United States of America; National Research Council, ITALY

## Abstract

Sleep is critical for memory consolidation, although the exact mechanisms mediating this process are unknown. Combining reduced network models and analysis of *in vivo* recordings, we tested the hypothesis that neuromodulatory changes in acetylcholine (ACh) levels during non-rapid eye movement (NREM) sleep mediate stabilization of network-wide firing patterns, with temporal order of neurons’ firing dependent on their mean firing rate during wake. In both reduced models and *in vivo* recordings from mouse hippocampus, we find that the relative order of firing among neurons during NREM sleep reflects their relative firing rates during prior wake. Our modeling results show that this remapping of wake-associated, firing frequency-based representations is based on NREM-associated changes in neuronal excitability mediated by ACh-gated potassium current. We also show that learning-dependent reordering of sequential firing during NREM sleep, together with spike timing-dependent plasticity (STDP), reconfigures neuronal firing rates across the network. This rescaling of firing rates has been reported in multiple brain circuits across periods of sleep. Our model and experimental data both suggest that this effect is amplified in neural circuits following learning. Together our data suggest that sleep may bias neural networks from firing rate-based towards phase-based information encoding to consolidate memories.

## Author summary

We show that neuromodulatory changes during non-rapid eye movement (NREM) sleep generate stable spike timing relationships between neurons, the ordering of which reflects the neurons’ relative firing rates during wake. Learning-dependent ordering of firing in the hippocampus during NREM, acting in tandem with spike timing-dependent plasticity, reconfigures neuronal firing rates across the hippocampal network. This “rescaling” of neuronal firing rates has recently been reported in multiple brain circuits across periods of sleep. Together, our results suggest that the brain is remapping frequency-biased representations of information formed during wake into timing biased-representations during NREM sleep.

## Introduction

How the brain binds various sensory features of life events into a neural representation for memory storage is a long-standing question in neuroscience. Available data suggest that initial memory encoding is driven by increases in activity (rate coding) among a specific population of neurons, often referred to as “engram neurons” [1–5]. Over time, these mnemonic representations are incorporated into more widely distributed networks in a process referred to as systems memory consolidation [6]. Recent data suggest that engrams are initially formed from heterogeneous neuronal populations with log-normal distributions of firing rates ranging over several orders of magnitude [7,8]. This heterogeneity may reflect populations encoding different aspects of experience [8–10], and which exhibit different dynamics during memory consolidation. A critical unanswered question is how these heterogeneous populations, distributed across the brain over vast synaptic distances, cooperate in the process of long-term memory storage. Recent experimental findings show that specifically during NREM, slow oscillations and sharp waves and ripples associated with them, promote temporal segregation between neurons with high intrinsic firing rates during wake and those firing less frequently, with the former leading the latter [7,11]. However dynamical processes mediating this segregation remain unknown.

At the same time slow oscillatory patterning of neuronal firing during sleep has been implicated in promoting synaptic plasticity and memory storage [12–19]. Network oscillations that are present in brain circuits during sleep have been implicated in promoting STDP by precisely timing the firing between pairs of neurons [14,15]. Hypothetically, the transition to oscillatory dynamics between wake and sleep could constitute the change in bias between rate coding (useful during initial learning) and phase coding (which may promote consolidation) [20]. For the reasons outlined above, this change in bias from rate to phase coding (i.e. when neurons’ respective phase of firing on each cycle conveys the information about the internal dynamical network relationships) may be promoting long-term information storage in brain networks.

Here, we combine computational modeling of a highly reduced neural network with analyses of *in vivo* recordings from CA1 obtained after contextual fear conditioning in freely behaving mice, to investigate how hippocampal network structure and dynamics are affected during contextual fear memory (CFM) consolidation following single-trial contextual fear conditioning (CFC) [17,21– 23] and pinpoint processes responsible for observed changes. This recent *in vivo* work has shown CFM consolidation is disrupted when CA1 oscillations (or CA1 network activity per se) are suppressed during post-CFC sleep [16,17,23,24]. Conversely, CFM can be rescued from disruption caused by experimental sleep deprivation when oscillations are driven optogenetically (via rhythmic activation of fast-spiking interneurons) in CA1 [17]. Further, the experimentally observed memory consolidation is associated with increased stability of temporal neuronal representations as measured by FuNS [25].

We show that regulation of neuronal excitability, mediated via changes in acetylcholine (ACh) muscarinic signaling analogous to those occurring in NREM sleep, drives changes in oscillatory properties in the CA1 network model, recreating a number of experimentally-observed phenomena. Within the model network, increases of stability are state-dependent (occurring in a state analogous to NREM sleep) and mediated by initial learning during stimulus exposure (modeled here as selective connection strengthening or increase in mean external drive to the neurons), associated with augmented post-learning network oscillations and blocked by suppression of inhibitory interneuron activity. Further, we show that the experimentally observed post-consolidation frequency dependent spiking frequency change, is mediated with a switch between rate coding (during a wake-like state) and phase coding (during a NREM-like state) in the network happening in tandem with STDP. Namely, NREM phase coding drives STDP between network neurons which causes dramatic, differential changes in the strength of reciprocal connections between highly active vs. sparsely firing neuronal populations. These changes lead to differential changes in firing rate in the two populations.

Together, our results show NREM-like state associated with low ACh conditions drives network dynamics to change bias between information coding schemes–a transition which occurs naturally through the sleep-wake cycle. Through this switch, functional network structures associated with engrams become more stable and robust for long-term information storage.

## Results

### Introducing a memory via heterogenous synaptic strengthening or increase in external drive, augments oscillatory dynamics and stabilizes functional connectivity patterns during simulated NREM sleep but not wake

We used a highly reduced *in silico* model network (with generic features) to determine whether ACh dependent muscarinic pathway may drive changes in network dynamics at the transition from wake to NREM sleep, in a universal way. We compared the in silico results with experimental recordings from hippocampal area CA1 following single-trial contextual fear conditioning (CFC) [24,26]–an experimental model system to investigate how network activity changes during memory encoding affects subsequent network dynamics. We analyzed both *in silico and* experimental *in vivo* recordings from CA1 to determine how functional network dynamics were affected by *de novo* memory formation [21,23].

ACh is known to regulate intrinsic neuronal membrane excitability, primarily through regulation of muscarinic receptors—when opened these receptors activate slow, hyperpolarizing potassium current. These receptors [27] are also present in CA1 [28–31].

In isolation, during wake-like state, high ACh blocks this current in excitatory neurons, which increases firing frequency responses to excitatory input (i.e. steepness of the Input-Frequency curve) and decreases neuronal propensity to synchronize which is mediated by the shape of the phase response curves (PRC) (i.e., type 1 excitability) [32,33].

Consequently, during wake-like state (high ACh, low conductance of m-current, *g*_*Ks*_), neurons behave as integrators to the incoming stimuli, responding to changes in input by sharply modulating their firing frequency (steep input-firing frequency (IF) curve). Thus, in this regime the neurons respond to the magnitude of their (external or network) input by modulating their frequency [34]. On a network level, this results in highly heterogenous neuronal firing frequencies and reduced synchronizabilty of neuronal spiking.

In contrast, low ACh during NREM sleep-like state allows slow potassium current (m-current) to play a larger role in membrane excitability, leading to spike frequency adaptation, reduced spike frequency response gain (i.e., a flat IF curve), subthreshold oscillations at theta band frequency, increased synchronization capacity (i.e., type 2 excitability) [33,32,35] and emergence of slowly moving waves of excitation resembling slow waves [36]. In this case, the neurons tend to spike with similar frequency across wider range of inputs, and lock to intrinsic or extrinsic oscillatory drive, especially if the oscillatory drive occurs at a resonant frequency. This leads to emergence of large-scale slow oscillations, where the magnitude of cell input is coded via relative phase of firing of neurons rather than frequency [34].

To investigate mechanisms involved in sleep-dependent aspects of memory consolidation, we simulated a reduced neural network model composed of excitatory principal neurons and inhibitory interneurons. For cells in the model, we used a conductance-based formalism (see **Methods**) incorporating a slow-varying potassium current which acts as a control parameter for neuronal firing dynamics [33]. Here, sleep/wake dynamics are mimicked by switching excitatory neurons from type 2 membrane excitability (NREM sleep) to type 1 excitability (wake), resulting in neuronal excitability change from integrator type response during wake, into resonator type response during NREM sleep. Since the muscarinic response of interneurons to ACh is complex and heterogeneous [37,38], we set inhibitory interneurons in the model to exhibit consistent type 1 dynamics (although permitting type 2 dynamics in interneurons yielded similar results).

Thus, here the “memory” is represented by a configuration of input patterns (either external to the network [applied via external current, *I*_*ext*_; see **Methods**] and/or internal ones driven by reciprocal synaptic connectivity), which alter the neurons’ relative frequency of firing during wake-like dynamics, or their relative phase relationships during NREM-like dynamics of the network.

Here we focused on pattern consolidation during NREM-like state in response to the memory trace being acquired during wake-like state. The predictions stemming from this consolidation in the model were closely compared with the *in vivo* data. To this effect, within the model we divided information storage into two phases. In the first, activation of subset of engram neurons by external input (during wake) results in rapid strengthening of connections between them—a process we refer to as “initial storage” hereafter. This process results in strengthened connections between this subset to cells (i.e. network heterogeneity), in the second phase, off-line network reorganization and consolidation is driven by STDP in a NREM-like state—which we refer to as “NREM dependent consolidation/reorganization”.

Critically, the recurrent excitatory connections within CA1 may be relatively few compared to other structures (although this is a matter of debate) [39,40]–which could limit the usefulness of our reduced model with more generic synaptic connectivity. To verify that these findings generalize to a network without substantial recurrent excitatory connections, we repeated these experiments in a model network without excitatory-to-excitatory connections but with modulating external excitatory input, *I*_*ext*_, to the excitatory network neurons. In this scenario, which would mimic learning-associated changes to CA1 excitatory input from CA3 alone.

**Fig 1:**
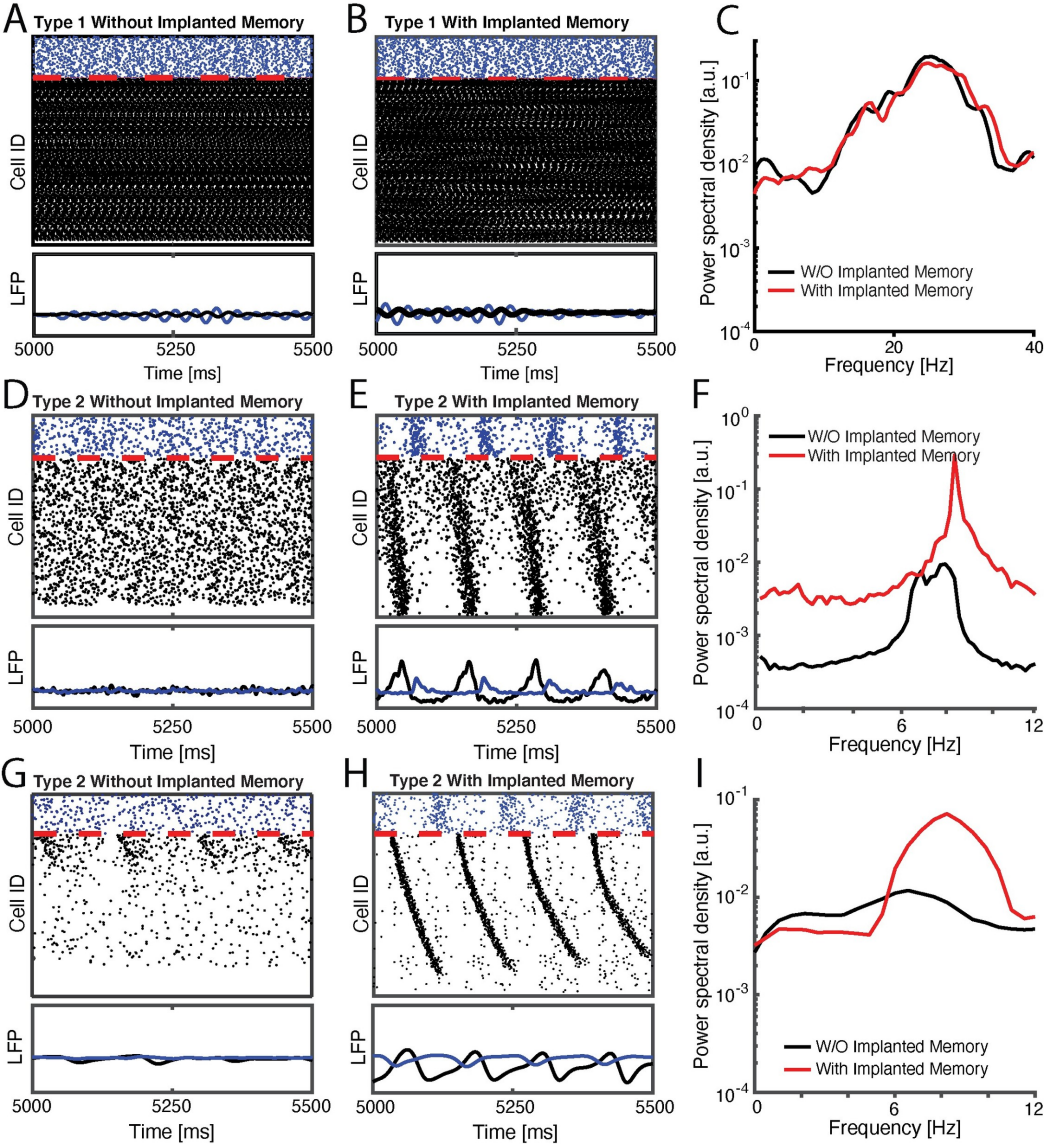
Type 2 model networks respond to sparse strengthening of excitatiory synapses or increasing excitatory input, through emergence of low-frequency rhythms and phase-locking. **SIMULATED DATA:** Raster plots (top) and cumulative signal (bottom) generated by inibitory (blue), and excitatory (black) neurons in a wake-like (high-ACh) state before (A) and after (B) initial memory storage. C) Fourier transform of the wake-like excitatory network signal before (black) and after initial memory storage (red) reveal enhanced beta/gamma oscillations. (**D, E**) raster plots (top) and cumulative signal (bottom) of NREM-like (low-ACh) state before (**D**) and after (**E**) initial storage (i.e. 20 fold strengthening of recurrent synapses between pairs of 25% of excitatory engram neurons). F) Fourier transform of the NREM-like excitatory network signal before (black) and after initial memory storage (red) reveal enhanced slow oscillations in theta range. (**G, H**) raster plots (top) and cumulative signal (bottom) of NREM-like (low-ACh) state in a network with removed reciprocal excitatory synapses, before (**G**) and after (**H**) initial storage (i.e. 5% increase in the mean external drive, *I*_*ext*_, to excitatory engram neurons). I) Fourier transform of the NREM-like excitatory network signal before (black) and after initial memory storage (red) reveal enhanced slow oscillations in theta range. On all rasterplots neurons are order as a function of their frequency in wake-like state.

Within this framework, we first investigated how strengthening excitatory synaptic connections between a limited subset of neurons, or changes in the external drive, *I*_*ext*_, events analogous to initial learning, affect network activity patterns during subsequent NREM sleep (i.e. low ACh/high *g*_*Ks*_ dynamics). Fig 1 depicts examples of raster plots, simulated LFPs, and their Fourier transforms for three cases: 1. when the network dynamics simulates waking state (i.e. high ACh dynamics, Fig 1A–1C), 2. When network dynamics simulates NREM sleep state, with memory encoded via strengthening of existing excitatory-to-excitatory connections (i.e. low ACh state, Fig 1D–1F), and 3. when network dynamics simulates NREM state, with memory encoded via modulation of the mean external input to the excitatory cells (Fig 1G–1I). Comparing raster plots (**top)** and simulated local field potentials (LFPs; **bottom**) for the network when excitatory neurons exhibit type 1 (wake; Fig 1A and 1B) and type 2 (NREM, Fig 1D and 1E) dynamics, before vs. after initial storage, reveals the emergence of well-defined slow oscillations (which are evident in periodic firing patterns of both excitatory and inhibitory neurons in the network) only in NREM-like state (Fig 1E) but not wake-like state (Fig 1B). In addition the wake-like network state exhibits oscillatory response in high beta/low gamma oscillatory range (Fig 1C). Thus only during NREM-like state does the initial storage lead to an increase network-wide low frequency spectral power (Fig 1F), consistent with previous *in vivo* work [17,23]. The neurons on the y-axis of the plots were ordered so that excitatory neurons receiving the highest excitatory drive were placed on the top and the ones receiving the lowest excitatory drive were place on the bottom. Please note the order of firing within the oscillatory burst, with the neurons receiving larger excitatory input firing first and the neurons receiving smaller excitatory input firing relatively later within the same burst.

From dynamical perspective this phenomenon is easy to explain and universal across biological and physical systems [41]. It is a well-established phenomenon that oscillators exhibiting somewhat different natural firing frequency lock with a phase-shift where intrinsically faster oscillators lead while slower oscillators lag. Here this natural firing frequency is input dependent.

Similar effect is observed when excitatory-to-excitatory connections are missing, and the memory is encoded via strengthening mean external input to the excitatory cells (Fig 1G–1I). Here however the mechanism is somewhat different, since synchronization cannot be supported via (non-existent) excitatory-to-excitatory connections. The external inputs interacting with subthreshold membrane oscillations trigger excitatory burst, with the relative phase of spiking of a given cell dependent of the magnitude of this input. This burst in turn activates inhibitory burst which resets the excitatory cells for the next cycle.

We note that the frequencies obtained for slow oscillations are primarily in theta band, whereas the frequencies observed in NREM sleep are usually lower [20]. This is due to highly reduced nature of the model, as here the detailed differences in peak frequency between the model and *in vivo* recordings are due in part to specific cellular resonance properties of neurons in the model network [14]. We show in S1 Fig that this frequency can be modulated via, for example, inhibitory network connectivity strength and time constant of activation of inhibitory postsynaptic potentials (IPSPs) associated with additional membrane currents which this model does not take into account.

The critical component here that drives the observed phenomena is mediated via homogenization of spiking frequencies and switch to type II dynamics regulated by activation of m-current due to low ACh levels, that in turn results in temporal organization of the network burst. Thus while resonant frequencies may vary by circuit, we hypothesize that this enhancement of coherent network oscillations (which has been widely reported in both human subjects and animal models following learning) [15] could drive STDP-based information storage in the network.

### NREM hippocampal network stabilization in vivo predicts successful fear *memory consolidation*

Next, we investigated how the magnitude of synaptic strength modifications, or the magnitude of changes of external drive to excitatory cells, taking place during initial learning subsequently affects functional network stability (FuNS) and network oscillations during sleep. We measured these two quantities as a function of strength of reciprocal excitatory connections between engram neurons, the number of connections that are being strengthened, and separately, in absence of excitatory-to-excitatory connections, as a function of mean external drive, I_*ext*_, to excitatory cells. Here, FuNS measures stability of functional connectivity between neurons, using the functional network stability metric (FuNS; see **Methods**) [25].

**Fig 2:**
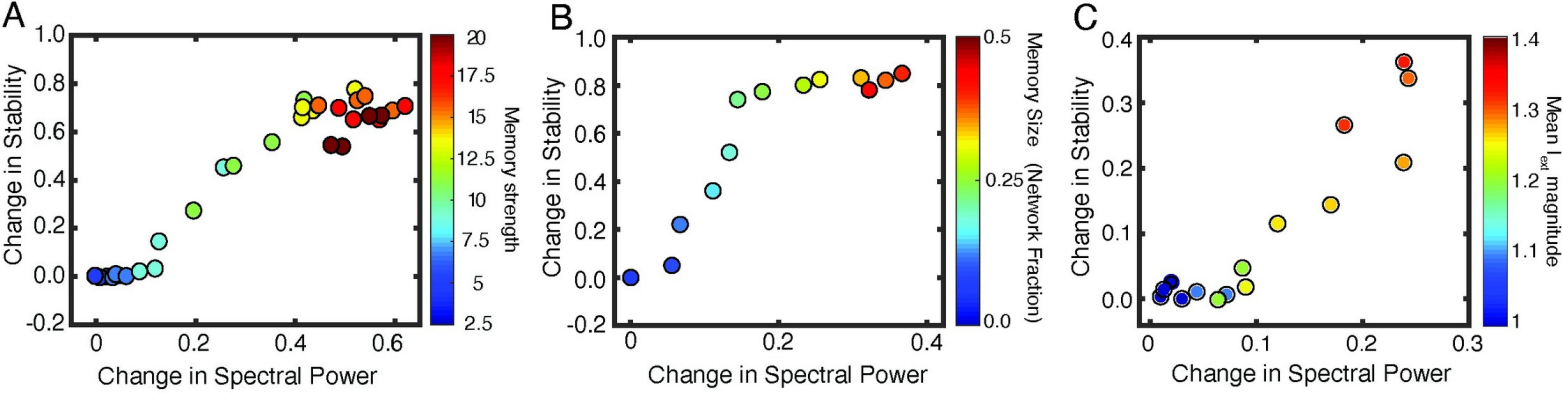
Memory-driven changes in network stability. Change in functional network stability (FuNS) and spectral theta power in response to strengthening of subset of reciprocal excitatory-to-excitatory connections (A) (color scale corresponds to synaptic multiplier), (B) different number of cells undergoing initial synaptic strengthening (color scale corresponds to fraction of cells that had strengthened incoming synapses), C) change in mean external current, *I*_*ext*_, received by excitatory neurons in absence or reciprocal excitatory-to-excitatory connections. In A the fraction of network with synapses strengthened was 25%; in B the strengthening multiplier was set to 20.

First, *in silico*, we simulated networks in NREM-like state (low Ach, high *g*_*Ks*_), with differential exposure to initial storage (i.e. strengthening reciprocal excitatory connections among subset of engram neurons as described above (Fig 2A), increasing the subset of neurons that are exposed to such a strengthening (Fig 2B), or increasing mean external drive, *I*_*ext*_, to the excitatory cells (Fig 2C)). We measured change in theta band power and functional network stability (FuNS; see methods) as a function of synaptic strengthening within the subset of engram cells (Fig 2A) and the number of cells undergoing this strengthening (Fig 2B) or magnitude of mean *I*_*ext*_ (Fig 2C). With increasing memory strength as well as the sub-group size, we observed rapid increase in both FuNS and oscillatory power in the model network (Fig 2A and 2B). This suggests that learning in the network directly augmented both network oscillations and stabilization of functional connectivity patterns in subsequent NREM sleep. While the multiplier magnitude may seem unrealistically large one needs to consider the size of the network and sparsity of these connections in the model. With the increased size/connectivity the multiplier will not need to be as high to achieve the same effect.

We have also observed significant increase in both FuNS and oscillatory power in the model network, when the mean value of *I*_*ext*_ was increased (Fig 2C). However, the mean change in stability was not as pronounced for higher levels of *I*_*ext*_ as when excitatory-to-excitatory connections were present in the network. That leads us to hypothesize that existence of even sparse excitatory-to-excitatory connectivity within CA1 increases stabilization of the output pattern of excitatory cells.

**Fig 3:**
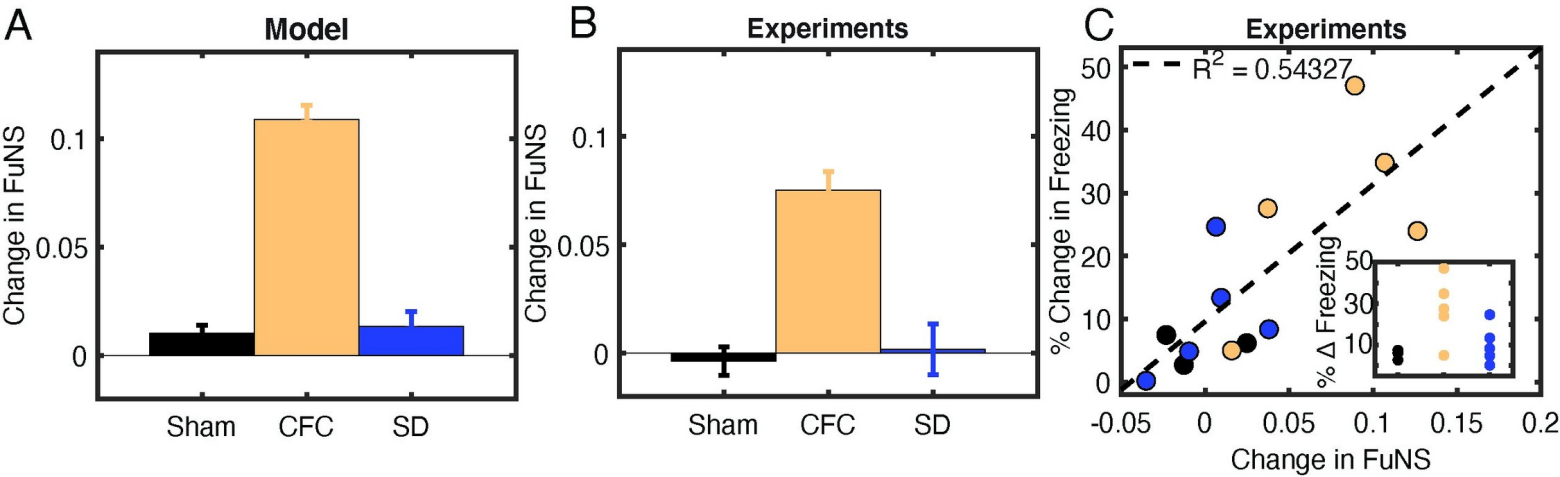
Functional network stability during NREM after initial exposure predicts future level of memory consolidation. **A) SIMULATED DATA:** Model predictions for the change in FuNS in each simulation group: Sham (NREM states without learning; n = 5) and SD (Wake states with learning; n = 5) show only marginal changes in FuNS whereas CFC (NREM states with learning; n = 5) show a maximal increase in FuNS. All error bars represent the standard error of the mean. **B) EXPERIMENTAL DATA:** FuNS analysis of CA1 recordings following CFC or Sham conditioning (Sham conditioned—black [n = 3], CFC + SD—blue [n = 5], CFC + sleep–yellow [n = 5]). *Ad lib* sleep post conditioning leads to the greatest increase in FuNS. **C) EXPERIMENTAL DATA:** Change in FuNS for each mouse after CFC predicted its memory performance 24 h later (% freezing; raw values shown as **inset**). Line indicates best fit to data with R^*2*^ = 0.54.

Next we compared our modeling results with the experimental data. To model effects of post-learning sleep deprivation (SD; high ACh, low *g*_*Ks*_), excitatory neurons were set to type 1 excitability in the presence of an implanted memory. To model interactions between learning and subsequent sleep, NREM (low ACh, high *g*_*Ks*_) was mimicked by setting excitatory neurons to type 2 excitability, in the presence or absence of initial connection strengthening (Learning and Sham, respectively). When the initially strengthened engram was present in networks with type 2 excitability, the network exhibited more stable network dynamics over time (Fig 3A). Conversely, type 2 “sham” networks (i.e. without initially strengthened engram) and type 1 networks with strengthened engram showed no change in stability.

We next tested how these features are affected during sleep-dependent consolidation of CFM *in vivo*. Mice either underwent single-trial CFC (placement into a novel environmental context, followed 2.5 min later by a 2-s, 0.75 mA foot shock; n = 5 mice), sham conditioning (placement in a novel context without foot shock; Sham; n = 3 mice), or CFC followed by 6 h of sleep deprivation (SD; a manipulation known to disrupt fear memory consolidation [17,22,42]; n = 5 mice). We measured changes in FuNS in these recordings after each manipulation by quantifying FuNS on a minute-by-minute basis over the entire pre- and post-training 24-h intervals and calculating their respective difference within each animal. Consistent with previous findings [21], we observed a significant increase in FuNS over the 24 h following CFC during NREM sleep (Fig 3B). In contrast, no change in NREM FuNS was seen in Sham mice or following CFC (during recovery NREM sleep, which is insufficient for CFM consolidation) in SD mice.

Group differences in NREM FuNS were reflected in the behavior of the mice 24 h post-training, when context-specific fear memory was assessed (inset Fig 3C, inset). Mice allowed *ad lib* sleep following CFC showed significantly greater freezing behavior when returned to the conditioned context than did Sham or SD mice. Moreover, CFC-induced changes in NREM-specific FuNS for individual mice predicted context-specific freezing during memory assessment 24 h later (Fig 3C). Thus, successful consolidation of a behaviorally-accessible memory trace *in vivo* is accompanied by increased NREM FuNS in the CA1 network.

Together, these results led us to hypothesize that oscillatory patterning could promote successful STDP-based consolidation of a hippocampal memory trace. We focus on this phenomenon in the following sections.

### Temporal organization of firing in network oscillations is a predictor of sleep-dependent firing rate reorganization via STDP

A series of recent studies have demonstrated that neuronal firing rates are renormalized across a period of sleep, with highly active neurons in a circuit reducing their firing rates, and sparsely firing neurons increasing their firing rates [7,8,43]. Sleep is essential for these firing rate changes, which do not occur across a period of experimental sleep deprivation [8]. We hypothesized that this phenomenon results from STDP driven by neurons phase-locking their firing to NREM sleep oscillations. Specifically, we predict that neurons that are highly active during wake will fire at an earlier phase within an oscillation than neurons with sparser firing.

**Fig 4:**
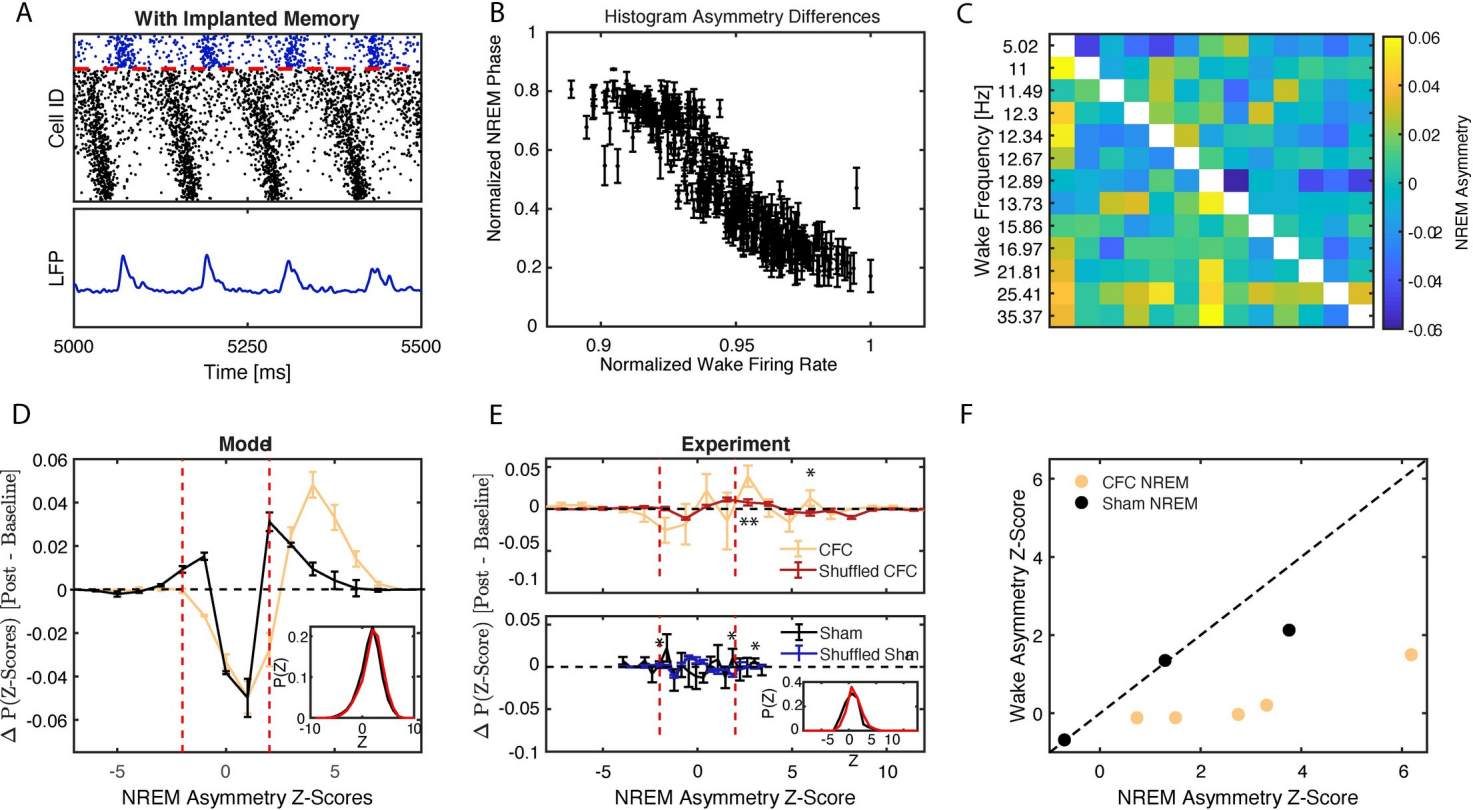
Mapping relative firing frequency distributions during wake onto firing phase relationships during NREM sleep. **A) MODEL DATA:** Calculation of mean phase of firing relative to oscillatory signal generated by inhibitory population; peaks in the inhibitory signal are used as the starting points of the phase calculation. **B) MODEL DATA:** Normalized phase of firing in NREM versus normalized wake frequency of excitatory neurons reveals that the neurons firing with the highest frequency align with an earlier phase of the excitatory network population whereas slower firing neurons align with later phases. ***C-F)*** Analysis of NREM firing asymmetry reveals enhanced wake frequency-dependent temporal relationships between neurons after learning. **C) EXPERIMENTAL DATA:** Formation of a pairwise firing asymmetry matrix of CA1 neurons’ activity recorded from a representative mouse during post-CFC NREM. Rows and columns have been arranged by wake frequency; color denotes pairwise firing asymmetry as defined in the **Methods** section. **D) MODEL DATA:** Weighted average of differences in distributions (see inset for a representative example scenario; black—baseline, red—post) of pairwise Z-scores for models with type 2 dynamics and learning (yellow) and with type 2 dynamics and no learning (black). **E) EXPERIMENTAL DATA:** Weighted average of differences in distributions (see inset in bottom panel for example of representative mouse; black—baseline, red—post) of pairwise Z-scores for NREM of CFC mice (top, yellow) and Sham mice (bottom, black), compared to differences in distributions calculated on random spike data following CFC (red, top) or Sham (blue, bottom) bursting architecture. **F) EXPERIMENTAL DATA:** Significance (Z-score) of global NREM network asymmetry vs. that of global wake asymmetry calculated for individual CFC (yellow) and sham (black) mice. Strong asymmetry is observed in NREM sleep but not in wake. Red dashed lines in (**D, E**) represent the significance cutoff of |*Z*|≥2. Error bars denote SE of repeated simulations (**D**) or SE across all experimental animals (**E**). * p < 0.05, ** p < 0.001 using a two-sided t-test.

To test this, we first calculated phase of firing of every excitatory neuron with respect to inhibitory LFP oscillations *in silico* (see Fig 4A and **Methods**). Fig 4B illustrates the relationship between model neurons’ phase of firing calculated during type 2 dynamics as a function of the normalized frequency during type 1 dynamics. We observed that the fastest firing neurons during waking (type 1) fire earlier in the phase of the excitatory network oscillation during NREM sleep (type 2). This suggests that neurons take on a phase-based, temporal coding strategy during NREM network oscillations which reflects differences in firing rate present during wake.

As previously mentioned, this is a universal phenomenon where generalized oscillators having faster natural frequency precede those with lower natural frequency when the two oscillators are coupled and their phase evolution is locked [41]. Here, during wake (high ACh state) the neurons exhibit type 1 dynamics—characterized by reduced capacity to synchronize [35], and relatively larger natural frequency differences (due to steepness of the Input current-spiking Frequency (I/F) curve) [34]. This effectively prevents neural synchronization with neurons that get higher input firing with higher frequency. Upon the switch to NREM like dynamics (low ACh state) the neurons switch to type 2 dynamics, which in contrast, is characterized by higher synchronizing capacity and reduced (but still present) natural frequency mismatch, due to flattened I-F curve. This dynamical state allows neurons to synchronize with the lead/lag pattern determined by the relative magnitude of the input individual neurons receive. Thus, as long as relative input patterns across wake sleep cycle stay the same the frequency during wake will be mapped onto the relative phase during NREM sleep.

To investigate whether this universal phase-locking phenomenon can be observed experimentally, we compared *in silico* and *in vivo* network dynamics during wake and during NREM, for CFC and sham situations. *In silico* sleep-dependent pattern consolidation was modeled through standard spike timing dependent plasticity (STDP) [44,45]. To that effect, we developed a metric to measure frequency-dependent phase-of-firing relationships between pairs of CA1 neurons by quantifying their spike timing asymmetry within bursts of activity (for a full description, see **Methods**). Briefly, in CFC and Sham hippocampal recordings, we first detected network bursts of firing across CA1 during NREM sleep. Within these bursts, we calculated the frequency dependent firing asymmetry between neurons–i.e. whether, statistically, neurons which fire faster during wake also show more advanced (i.e., leading) firing phase during NREM sleep. Namely, for each recording, we defined an asymmetry matrix *A*, an *N*×*N* matrix whose rows and columns were ordered by the relative firing rate of neurons during wake (Fig 4C; see **Methods** for details).

We determined the significance, *Z*_*ij*_, of asymmetry for every pair, *A*_*ij*_, via bootstrapping and compared the normalized histograms of *Z*_*ij*_ (see representative examples from simulated data, Fig 4D inset) from a network undergoing STPD when: 1) an engram was partially strengthened (initial storage—model CFC, Fig 1D) and 2) when it was not (model sham, Fig 1C). The distributions show stronger skewing towards significant (Z > 2) asymmetry pairs after NREM state STDP (with respect to baseline dynamics), for CFC-like engram strengthening (Fig 4D–yellow line) in a sham (no engram strengthening) condition (Fig 4D–black line).

We next compared *in silico* results to the experimental distributions obtained from wake and NREM for CA1 recordings from experimental CFC and Sham groups, and found similar results (Fig 4E). Fig 4E, shows the experimental differences between baseline and post-learning distributions for CFC (Fig 4E, top panel) and Sham (Fig 4E, bottom panel). Significance values for these distributions were calculated by comparison with randomized distributions (“shuffled CFC” and “shuffled sham”), where the bursting architecture between non-randomized and randomized sets was conserved (see **Methods**). Greater significance occurs for positive Z-values following CFC (Fig 4E; top—yellow line) as compared to sham (Fig 4E; bottom—black line), indicating that there is a shift in asymmetry during fear memory consolidation.

These distributions qualitatively resemble the ones obtained *in silico* (Fig 4D) and show that consolidation after CFC increases the number of neuron pairs with consistently significantly asymmetric firing patterns (Z > 2) during NREM (Fig 4E; top—yellow line), as compared to sham (Fig 4E; bottom—black line). This indicates the presence of a phase-coding mechanism during NREM sleep, which is sensitive to neuronal and network activity changes caused by initial storage in wake.

To ensure that the shift in pairwise Z-score distributions is not a simple reflection of firing frequency differences between neuronal pairs in NREM, we measured global asymmetry across the network during NREM as a function of firing frequency during wake, for every animal separately. To do this we calculated a mean asymmetry score by subtracting the mean asymmetry of the upper triangle and lower triangle of the asymmetry matrix (Fig 4C). We then subjected the result to bootstrapping to estimate its significance. We next reversed the calculation and repeated the asymmetry calculation now for burst-ordering detected during wake, as a function of firing frequency during NREM (i.e. calculation that would indicate **reverse hypothesis**—relationship between **phase coding in wake** and **frequency coding in NREM**). These results are depicted in Fig 4F, where we plot the significance of NREM asymmetry vs. wake asymmetry. We observe that following CFC (but not sham conditioning), all animals have higher significances for NREM asymmetry as compared to the reversed wake asymmetry.

Based on this relationship, we hypothesized that due to the resulting firing phase relationships in NREM, STDP in this context would cause: 1) excitatory connections from high-firing to low-firing neurons to be strengthened, and 2) connections from low-firing to high-firing neurons to be weakened.

### Network oscillations promote temporal coding during NREM *sleep*

**Fig 5:**
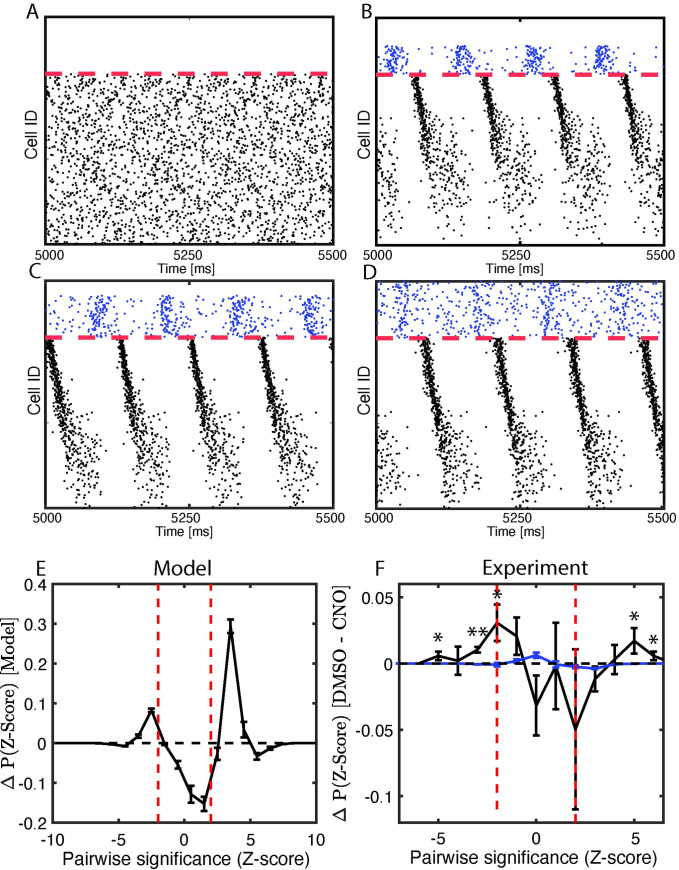
Disruption of network oscillations diminishes spike timing relationships between neurons. **A-E) MODEL DATA: A-D)** Simulated raster-plots; NREM-like network dynamics for the progressively larger disruption of inhibitory firing. **E)** Weighted sum of differences in model asymmetry significance distributions after consolidation (unperturbed model in (**D**) minus model without inhibition in (**A**)). **F**) **EXPERIMENTAL DATA:** Difference in experimental asymmetry significance distributions, shown as the black trace, for CA1 recordings for mice expressing the inhibitory DREADD hM4Di in PV+ interneurons, treated with either DMSO (analogous to the full model network) or CNO (analogous to reduced network inhibition); compare to randomized spike times used to estimate significance of these values, shown in blue. Error bars denote SEM of repeated simulations (E) or SEM across all experimental animals (F). * p < 0.05; ** p < 0.01 using a two-sided t-test.

We hypothesize that via STDP, network oscillations—which are naturally augmented during post-learning NREM sleep—play a crucial role in driving memory consolidation. To test this, we next investigated how disruption of network oscillations affects phase-coding mechanisms. In our model neural network, following the experimental manipulation [23] we first prevented a fraction of inhibitory neurons from firing in the network after initial storage (i.e. partial engram strengthening; Fig 5A–5D). We observe that disruption of progressively larger fraction of inhibitory neurons decreases the numbered of neurons entrained into oscillatory dynamics (Fig 5A–5D).

We next measured the effect that the complete disruption of inhibitory firing (Fig 5A) has on post-learning firing asymmetry (Fig 5E). Here we measured differences in distribution of asymmetry of Z-score values between the cases when inhibition was intact (Fig 5D) and fully disrupted (Fig 5A). With intact inhibitory neuron activity, the model exhibited significant positive firing asymmetry as compared to disrupted inhibition.

We also analyzed the firing asymmetry of CA1 recordings from mice expressing the inhibitory DREADD (Designer Receptor Exclusively Activated by Designer Drugs) hM4Di in parvalbumin-expressing (PV+) interneurons. These mice were treated with either a vehicle (DMSO) or the hM4Di activator clozapine-N-oxide (CNO, to activate hM4Di and suppress PV+ interneuron activity) immediately following CFC. Previous work has shown that CA1 network oscillations and CFM consolidation are both disrupted by post-CFC inhibition of PV+ interneurons [17,23]. We found that disruption of PV+ interneuron activity with CNO reduced firing rate-associated firing phase asymmetries during post-CFC NREM network bursts relative to DMSO-treated mice, which have normal CA1 oscillations and CFM consolidation (Fig 5F, black trace). Similar to what was done for CFC vs. Sham data (Fig 5D), we compared Z-Score distribution to another based on randomized spike times within bursts following DMSO vs. CNO architecture (Fig 5F, blue trace); we find that the peaks at positive extreme values of the distribution are significant, indicating a greater asymmetry shift in that direction. Thus, for both network models in a type 2 regime and the CA1 network during NREM sleep *in vivo*, disruption of interneuron-driven oscillations impairs temporal coding.

### Frequency-dependent NREM firing asymmetry effects network reorganization through STDP

**Fig 6:**
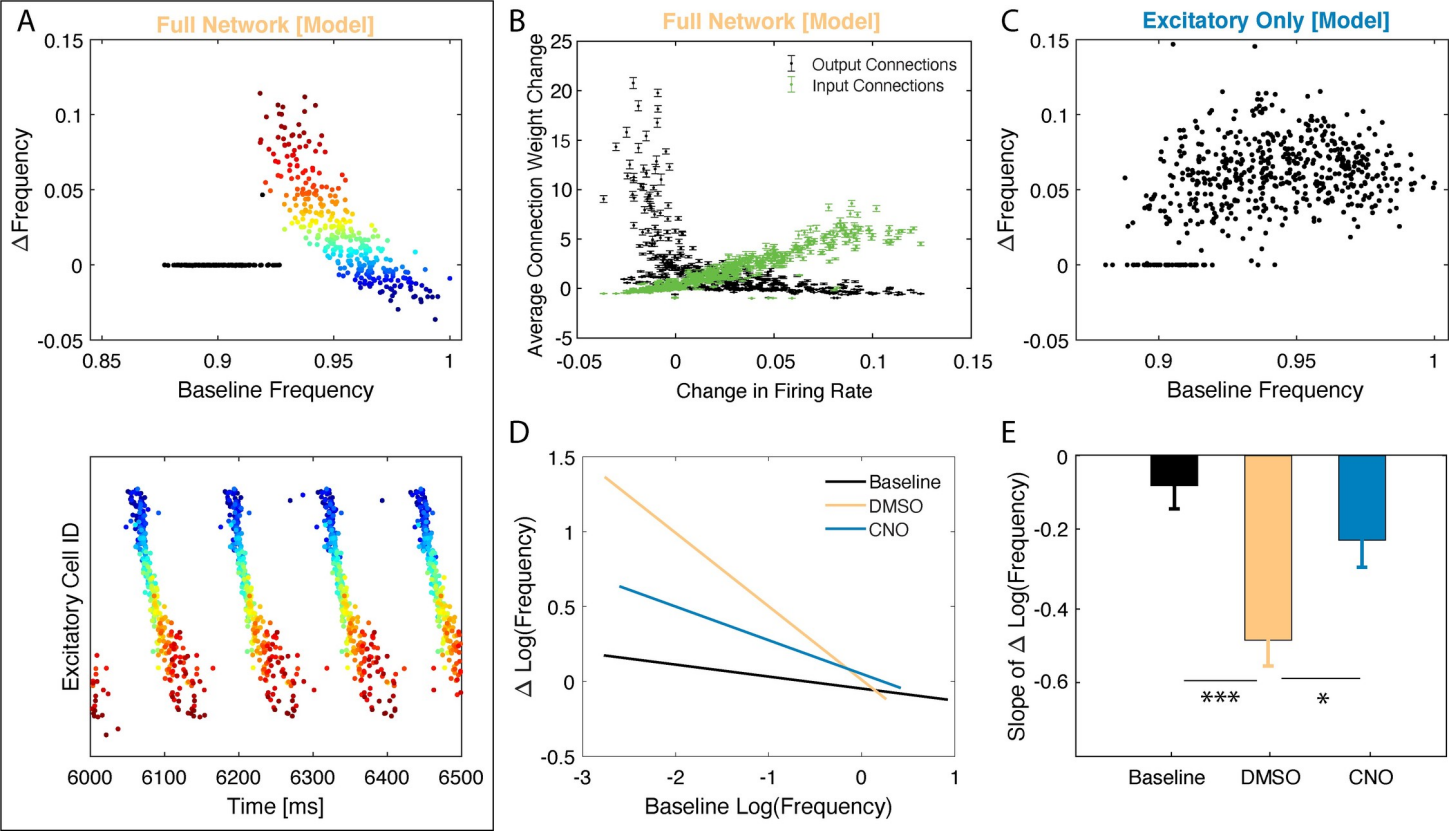
NREM dependent reorganization differentially affects frequency of firing neurons during wake-like dynamics–model prediction and experiment. **SIMULATED DATA**: **A) Top:** Changes in individual neurons’ firing frequency (normalized to baseline) observed during wake-like dynamics recorded across post-learning NREM sleep as a function of normalized baseline firing frequency (top). Conditions comparable to **Fig 5D.** Colors represent baseline frequency. **Bottom:** Snapshot of the corresponding raster plot in NREM sleep at the onset of consolidation. Neurons are color-coded based on their baseline frequency during wake like dynamics and the color is conserved in the raster plot. Black data points (top) are for neurons which did not fire during NREM sleep. Of the neurons that are consistently active, those with initially lower frequency increase their frequency whereas neurons with initially higher frequency decrease their frequency. **B)** Average connection weight change for a given neuron vs its frequency change during wake-like dynamics, observed in A. Neurons that increase their firing rate do so due to increased synaptic input, at the same time their mean strength of output connections decreases. **C)** Change in firing frequency (normalized to baseline) as a function of normalized baseline firing frequency in the absence of inhibition. Conditions comparable to **Fig 5A.** Unlike the full-network condition (with inhibition), firing frequency changes homogenously across baseline firing rates. **EXPERIMENTAL DATA**: **D)** Best fit lines of the change in log firing rates vs the initial log firing rate, comparing baseline recordings (solid lines; composite n = 11) to post recordings (dashed lines) for the first 6 hours post CFC for mice expressing the inhibitory DREADD hM4Di in PV+ interneurons, treated with either DMSO (Yellow; n = 3; analogous to the full model network) or CNO (Teal; n = 3; analogous to reduced network inhibition), and wild type mice with post-CFC SD (Blue; n = 5). **E)** Slope comparison of change in log firing rates for DMSO, CNO, and SD baseline and post-CFC recordings. Analysis of covariation revealed statistically significant slope differences between DMSO and CNO (* indicates p < 0.05), and DMSO and Baseline (*** indicates p < 0.0001).

We next examined whether firing rate reorganization occurs during NREM sleep via STDP in the context of firing asymmetries described in the sections above. First, we compared in silico firing rate reorganization between type 2 excitatory networks with and without firing in inhibitory neurons, when synaptic strength could evolve over time using an STDP-like plasticity rule. We examined the changes in wake neuronal firing frequencies after an interval in these two scenarios. In the model with normal inhibitory neuron firing, STDP-based synaptic changes in a type 2 (NREM) regime led to a simultaneous increase in the firing rates of principal neurons with the lowest baseline activity and decrease in the firing rates for the most active neurons (Fig 6A). We color-coded neurons based on their relative change in frequency across sleep (Fig 6A top). As shown in Fig 6 (bottom), neurons which fire faster (vs. slower) during baseline wake-like dynamics (i.e. high ACh, low *g*_*Ks*_; type 1 dynamics) also fire earlier (vs. later) in the oscillation NREM-like dynamics (i.e. low ACh, high *g*_*Ks*_; type 2 dynamics), consistent with Fig 4, and experience a decrease (vs. increase) in firing rate due to STDP. Some neurons did not fire during NREM and so did not show a firing frequency change due to STDP (black points in Fig 6A)–this represents selective cell recruitment into the memory engram. Here, this initial firing frequency-dependent frequency change is driven by changes of overall synaptic input (green) to the said cells (Fig 6B). The cells exhibiting decrease in firing frequency also exhibit overall decrease in their synaptic input, however their output (black) is significantly strengthened due to timing of firing asymmetries (Fig 4B). Disruption of network oscillations via inhibitory neuron silencing disrupted the relationship between firing rate changes across the type 2 regime and baseline firing rates for principal neurons (Fig 6C).

We compared these results with data recorded from the hippocampus of mice with and without DREADD-mediated disruption of PV+ interneuron activity [17,23]. We measured changes in firing frequency across a six-hour time interval at the start of the rest phase (i.e., starting at lights on), either at baseline (i.e., the day before CFC) or in the hours following CFC. Firing rate changes for each neuron were calculated for mice treated with either DMSO or CNO as a function of their baseline firing rate. The resulting best-fit lines (Fig 6D) reveal that while CA1 neurons show a relatively low degree of firing rate reorganization across baseline rest, following CFC reorganization is more dramatic. The greatest degree of reorganization is seen after CFC in the control (DMSO) condition, with less-dramatic firing rate changes seen in mice with disrupted CA1 PV+ interneuron activity (CNO) (Fig 6D and 6E).

Comparing the slopes of firing rate vs. firing rate change relationships in CA1 for post-CFC recordings (Fig 6E), we found significantly weaker reorganization of firing rates in CNO-treated mice relative to DMSO-treated mice. This reorganization of firing rates across the network is an important prediction of the model, as it suggests a possible universal network-level correlate of sleep-based memory consolidation *in vivo*.

Here we have used a standard asymmetric STDP rule, where synapses are strengthened when presynaptic neuronal firing leads firing in the postsynaptic neuron, and are weakened when presynaptic neuronal firing follows firing in the postsynaptic neuron. However, in some circumstances STDP rules can vary, leading to coincident firing leading to either potentiation or depression, regardless of order of firing [1]. An unanswered question is how the network would be altered by state transitions with various STDP conditions, thus we tested the effects of NREM sleep with potentiation-only (S2 Fig) and depression-only (S3 Fig) STDP rules. For potentiation-only STDP the relationship between initial firing rate and firing rate change is reversed—with initially faster-firing neurons firing even faster after a period of NREM sleep. With a depression-only STDP rule, the relationship is similar to that presented in Fig 5D, although all frequency changes are negative across NREM. As neither of these relationships is observed experimentally (Fig 5D), this suggests that asymmetrical STDP rules alone can explain firing rate changes across NREM sleep in CA1.

**Fig 7:**
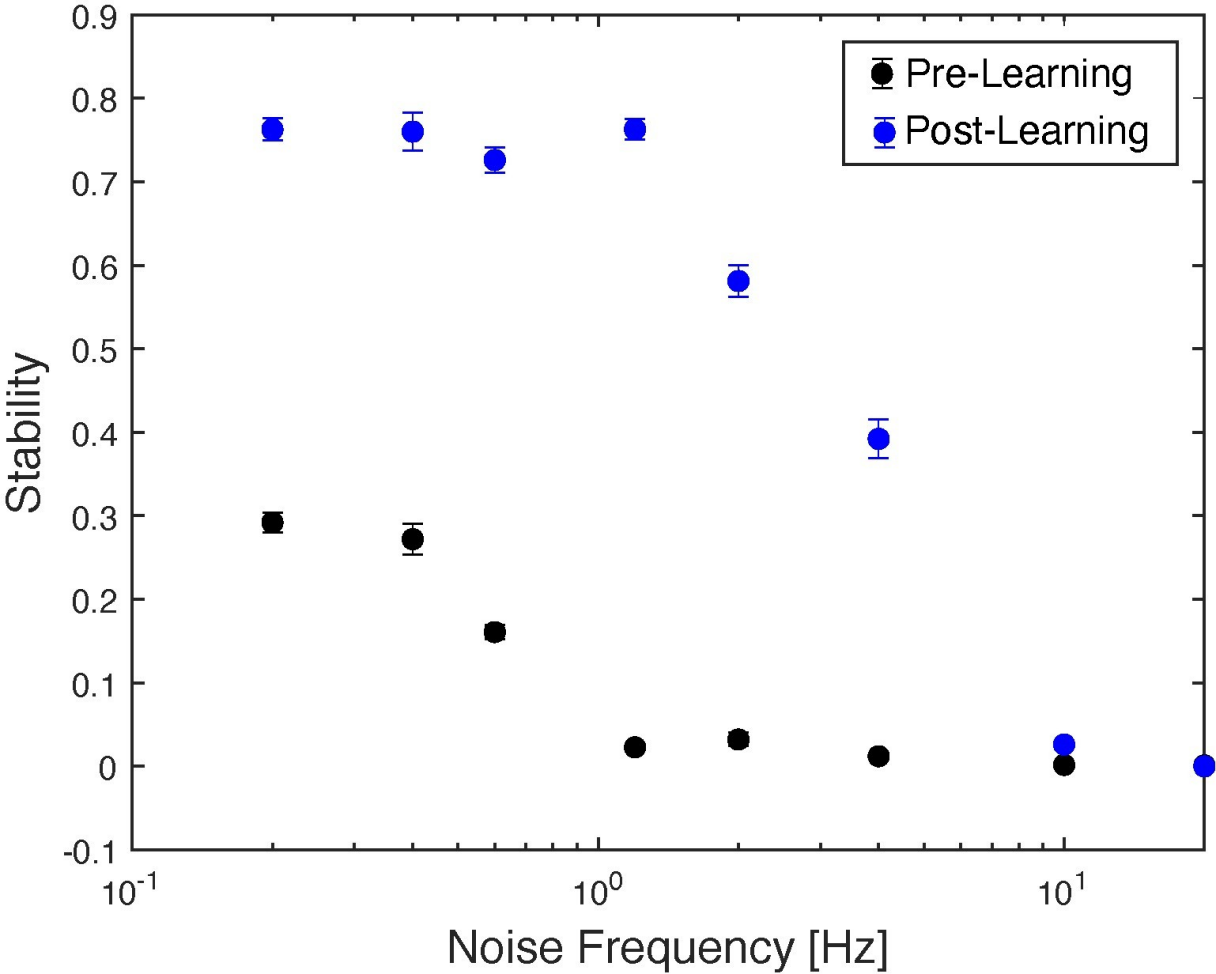
STDP driven NREM-like memory consolidation results in an increased stability of the network dynamics across wide range of noise levels in the network. Black dots–dynamics of the network prior STDP driven NREM-like memory consolidation. Blue dots—network dynamics post STDP driven NREM-like memory consolidation.

Finally, we set out to quantify effects of sleep-dependent consolidation. As mentioned before, the “memory” is represented by a configuration of input patterns (external to the network (*I*_*ext*_, see methods) and/or internal ones driven by synaptic connectivity, which exemplify themselves via relative frequency of firing during wake-like dynamics, or relative phase relationships during NREM-like dynamics of the network. FuNS measures the robustness of the of the functional network connectivity which is exemplified via stability (over time) of the locking of the firing patterns between the neurons. Thus, sleep-dependent consolidation of the memory should result in an increased FuNS as compared to the unconsolidated dynamics. We investigated FuNS for consolidated and unconsolidated network dynamics for different frequency of noise fed into the network (Fig 7). The stability of the consolidated (post-learning; Fig 7, blue dots) dynamics is significantly higher than that of unconsolidated one (pre-learning; Fig 7, black dots) except for the highest levels of noise when both cases are unstable.

## Discussion

Sleep is vital for successful memory consolidation across organisms and different types of memories (e.g., those mediated by network activity in hippocampus vs. sensory cortex [15]). Similarly, recent experimental advances have shown that oscillatory dynamics in neural circuits–associated with sleep—play a vital role in memory consolidation [23,46,47]. In hippocampus, CFM consolidation relies on *ad lib* sleep in the hours immediately following CFC [17,22]. Consolidation of CFM is associated with enhanced delta- (0.5–4 Hz), theta- (4–12 Hz), and ripple-frequency (150–200 Hz) hippocampal network oscillations in the hours following learning [23].

Here, we observe that, during NREM sleep, representation coding undergoes functional remapping from a frequency-based coding of information predominant during wake (and particularly, during learning) to a timing-based representation predominant during sleep. Further, we argue that temporal organization of neuronal firing by network oscillations expressed during NREM sleep promotes feed-forward synaptic plasticity (i.e., STDP) from highly active neurons to less active ones. This process in turn promotes recruitment of additional neurons into the engram–the “systems consolidation” underlying long-term memory storage.

Our reduced model demonstrates that changing ACh level during wake and NREM sleep can play here a key role as it mediates changes in neuronal excitability, leading to highly heterogenous in frequency, asynchronous network-wide spiking during wake, and homogenized in frequency, temporally locked network-wide spiking during NREM sleep. Specifically, we show that the initial encoding of memories in a network (e.g. in the CA1 network during CFC) augments low frequency network oscillations (Figs 1 and 2). The augmentation of these oscillations occurs in a NREM sleep-like, low-ACh, type 2 network state. In this state, neurons are less responsive to input (i.e. the input-frequency curve flattens [14]), and they exhibit higher propensity to synchronize to periodic input (i.e., network oscillations). This locking to network oscillations leads to more stable firing relationships between neurons. This may occur via strengthening of excitatory-to-excitatory connections in the network, or if they are not available via increased external input from other modalities (e.g. CA3 in hippocampus). We observe this increase in functional network stability (i.e. increase in FuNS) both in our hippocampal network model after introduction of either, a synaptically-encoded memory or via increasing external input (Fig 2), and in the mouse hippocampus *in vivo* following single-trial CFC (Fig 3).

Our previous and current data show that sleep-associated FuNS changes in CA1 following CFC is a salient feature of network-wide dynamics that accompanies successful memory consolidation (Figs 3–6) [8,17,21,23]. We find that increased FuNS is associated with stronger low-frequency oscillatory patterning of the network, and predicts which experimental conditions will support, disrupt, or rescue fear memory consolidation (Fig 3C). This increased stability, in turn, mediates mapping between firing rates during wake and relative phase-of-firing during NREM sleep (Fig 4A and 4B)–such that firing of neurons with higher baseline firing frequency leads those with lower baseline firing frequency. We showed that this frequency-vs.-timing relationship is also detected in experimental data recorded from CA1 during NREM sleep (Fig 4C, 4E and 4F). Moreover, when network oscillations are abolished by blocking inhibitory neuron activity (Fig 5) the frequency/timing relationship is disrupted, indicating that oscillations do play central role in this process.

We hypothesize that this mechanism allows for recruitment of diversely firing populations of neurons (with their firing frequencies distributed over 3 orders of magnitude) into the engram during sleep mediated memory consolidation, which is thought to be critical to the network operations underlying hippocampal function [7,48]. Homogenization of firing frequencies during sleep together with increased synchronization propensity and universal organization of the network bursts where intrinsically high firing frequency neurons lead the slow firing frequency ones, allows STDP-like mechanisms to recruit the low firing cells into the engram by linking them with the high frequency ones.

High-density electrophysiological recordings confirm that the most pronounced effect of sleep, and especially NREM sleep, on cortical firing rates is a narrowing of the firing rate distribution [7,43]. Other experimental findings found similar mean firing frequency dependent temporal ordering; Fernandez-Ruiz et al. [11] reported that the average rank order of a neuron’s within-ripple sequence was negatively correlated with that neuron’s baseline firing rate calculated from the entire sleep—wake session. More generally in was found that spiking during the NREM slow oscillation reveals an intrinsic temporal separation between high and low firing rate units, such that neurons with higher firing rates tend to spike before those with lower firing rates at the DOWN->UP transition [49].

Finally, when synaptic strength in the model is allowed to evolve through STDP, we observe this reorganization of firing rates across NREM sleep—with sparsely firing neurons increasing their firing rate substantially, and highly active neurons decreasing their firing rate. These results are also observed in CA1 during sleep-dependent CFM consolidation (Fig 6). We again observed a disruption of these effects when the oscillatory network activity is reduced via manipulations of interneurons. Similar sleep-associated firing rate changes have been reported in neurons recorded from various neural circuits [7,8,50]. Moreover, this network reorganization results in increased network stability indicating increased robustness of the memory trace (Fig 7) in terms of their temporal representation. This last result agrees with number experimental studies that found that neurons having widely different intrinsic firing frequencies act differentially within the activated engram and possibly play different coding roles. It was reported that such a activation sequence is composed of fast-firing subset of pyramidal neurons having low spatial specificity and limited activation change across sleep-experience-sleep cycle, and a slow-firing, highly plastic subset that elevated their association with ripples, and showed increased bursting and temporal coactivation during postexperience sleep [51,52]. Cognitively, these high activity principal cells were shown to form a core of each memory, with low activity cells joining as co-motives across the behavioral events [48].

Taken together, our results suggest a universal mesoscopic network mechanism underlying what is commonly referred to as systems memory consolidation. They also provide support for the hypothesis that while the brain may be biased towards firing rate-based code during waking experience, the increased bias towards firing phase-based information coding in the context of network oscillations in sleep could play an instructive role for memory storage. This mechanism would mitigate the aforementioned limitations of rate-based information coding in the brain [20]. At the same time, it is however clear that the two coding schemes are able to co-exist depending on specific spatio-temporal attributes of the cognitive tasks.

We also note limitations of the presented model. Here specifically, we do not consider sequential activation of place cells as animal traverses through different locations in an environment. These would naturally impose sequential activation also in the waking activity pattern. We have however shown previously that such sequences can be also represented and stored during low ACh states [14].

Our model also does not take into account the emergence of theta band oscillation observed during REM sleep–a high ACh state. We hypothesize that the mechanism generating these oscillations is different, resembling higher frequency PING-like [53] mechanism, with these oscillations playing very different cognitive role. It was found experimentally that during REM, unlike NREM, the firing frequencies become even more heterogenous [50] which could be caused by increased steepness of the I-F curve.

Finally, this, being highly reduced, model does not take into account number of known network, cellular and molecular pathways. Here we focus on highlighting only possible role of changing ACh levels to explain experimental data in a highly generic network. Because of this the frequencies of slow oscillations obtained in NREM are generally higher than those observed experimentally [20]. We show in S1 Fig that the specific frequency of the slow oscillations can be manipulated by changing connectivity properties in the model. While this is a clear limitation, we don’t believe that this shift in observed slow oscillation frequencies bears effect on the results shown here.

Finally, ACh is not the exclusive player in terms of the changes in neuromodulation at the transitions between wake and NREM sleep [54]. Adenosine for example, can directly regulate ACh release via A1 receptors targeting cholinergic centers, but also regulate the potassium current via G-protein inwardly rectifying potassium (GIRK) channels augmenting the described cholinergic effect. Adenosine agonists are known to decrease wakefulness and increase sleep, tend to increase deeper stages of SWS, and increase slow wave activity or delta power. Conversely, adenosine receptor antagonists increase wakefulness and decrease sleep [55]. It was shown in *in vitro* conditions, that adenosine enhance slow oscillations of single neurons in the absence of other modulatory input [55,56]. Understanding more closely the interactions, and differential roles of the two mechanisms would be a focus of another study.

While the present study is focused here on computational model to predict data from the CA1 network during CFM consolidation and highlight the role ACh can play in this process, we believe that the mechanisms outlined here may be universally true. For example, sleep, and sleep-associated network oscillations, are required for consolidation of experience-dependent sensory plasticity in the visual cortex [18,21,47], and disruption of other hippocampal oscillations during sleep disrupts consolidation of other forms of memory [12]. Moreover, similar frequency-dependent changes in neuronal firing rates are also observed across periods of sleep in the visual cortex [8] and frontal cortex [7]. Based on these and other recent data linking network oscillations in sleep to many forms of memory consolidation, this suggests a unifying principle for sleep effects on cognitive function, and one that could reconcile discrepant findings on how sleep affects synaptic strength [15]. It also provides an expanded and possibly alternative explanation of the role of sleep in memory management than what is often proposed [57]. Here we show that NREM sleep facilitates both increases and decreases in neuronal firing rates in the context of network reorganization, while at the same time recruiting heterogeneous neuronal populations during systems memory consolidation.

While the form of learning modeled here is not reliant on sequential neuronal activation during memory encoding, one possibility is that a similar mechanism may be associated with consolidation of memories for events during which neurons are sequentially activated [58–60]. Future studies will be needed to examine how temporal patterning of neuronal during post-learning NREM sleep relates to previously-studied “replay” of firing sequences reported in structures like the hippocampus during sleep following sequential spatial tasks. One possibility is that sequential replay presents a special case of NREM-dependent patterning of firing based on prior wake firing rates, as described here.

## Methods

### Ethics statement

All experimental procedures were approved by the University of Michigan Animal Care and Use Committee (animal protocol #: 00008333).

### Experimental design

#### Hippocampal recordings, fear conditioning, and sleep deprivation

Male C57BL/6J mice between 2 and 6 months were implanted using methods described previously [21,61]. Recording implants (described in more detail in [21]) consisted of custom built driveable headstages with two bundles of stereotrodes implanted in bilateral CA1 and three EMG electrodes to monitor nuchal muscle activity. The signals from the stereotrodes were split into local field potential (0.5–200 Hz) and spike data (200 Hz-8 kHz).

Following post-operative recovery, mice either underwent CFC (placement into a novel environmental context, followed 2.5 min later by a 2-s, 0.75 mA foot shock; n = 5 mice), Sham conditioning (placement in a novel context without foot shock; Sham; n = 3 mice), or CFC followed by 6 h of sleep deprivation by gentle handling (a manipulation known to disrupt fear memory consolidation [17,21,22,42]; SD; n = 5 mice). Spike data from individual neurons was discriminated offline using standard methods (consistent waveform shape and amplitude on the two stereotrode wires, relative cluster position of spike waveforms in principle component space, ISI ≥ 1 ms) [8,17,21,23,47]. Only neurons that were stably recorded and reliably discriminated throughout the entire baseline and post-conditioning period were included in subsequent analyses of network dynamics.

24 h following CFC or Sham conditioning, freezing behavior upon return to the conditioning context was measured to evaluate CFM.

#### Pharmacogenetic inhibition of interneurons

2-3-month-old male Pvalb-IRES-CRE mice were bilaterally injected with either the inhibitory receptor hM4Di (rAAV2/Ef1A-DIo-hM4Di-mCherry; UNC Vector Core: Lot #AV4,708) or a control mCherry reporter (raav2/Ef1A-DIo-mCherry; UNC Vector Core: Lot #AV4375FA) (methods further elaborated in [17]. Using the same implant procedures described above, the animals were implanted with stereotrode bundles.

After allowing 4 weeks for viral expression, the animals underwent contextual fear conditioning (as described above). Post-shock, mice were either given an i.p. injection of either 0.3 mg/kg clozapine-N-oxide (CNO) dissolved in DMSO (to activate the DREADD) or DMSO alone (as a control) [23].

### Computational modeling

#### Mixed excitatory-inhibitory conductance-based neuronal networks

Conductance-based neuronal networks containing both excitatory and inhibitory neurons were modeled using a modified Hodgkin-Huxley formalism [33,62]. The time-dependent voltage *V*_*i*_ of a single neuron is given by

$$C_{m}\frac{d}{dt}V_{i}=-I_{Na}-I_{K}-I_{K_{s}}-I_{leak}+I_{ext}-I_{Synaptic}+I_{noise}$$

where *C*_*m*_ is the membrane capacitance, *I*_*ext*_ is the fixed external input (DC) used to elicit spiking; *I*_*ext*_ ∈ [1.08,1.2] for excitatory cells and *I*_*ext*_ ∈ [−0.09,−0.08] for inhibitory cells; *I*_*leak*_ = 0.02(*V*_*i*_+60) is the leakage current, and *I*_*Synaptic*_ = (∑_*j*∈*Excitatory*_ *g*_*E*−*X*_*S*_*ij*_)(*V*_*i*_−*V*_*Excitatory*_)+(∑_*j*∈*Inhibitory*_ *g*_*I*−*X*_*S*_*ij*_)(*V*_*i*_−*V*_*Inhibitory*_) is the total summed synaptic input received by a neuron from its pre-synaptic partners and *g*_*I*−*X*_ and *g*_*E*−*X*_ represent the synaptic conductance for connections from inhibitory and excitatory neurons to their post synaptic targets *X* (values provided below). The synaptic reversal potentials are *V*_*Excitatory*_ = 0 *mV* and *V*_*Inhibitory*_ = −75 *mV*. Here, $S_{ij}=\exp\left(-\frac{\Delta t_{ji}^{spk}}{\tau_{s}}\right)-\exp\left(-\frac{\Delta t_{ji}^{spk}}{\tau_{f}}\right)$ represents the shape of the synaptic current, given the difference in spike timing between the post-synaptic neuron *i* and the recently fired pre-synaptic neuron *j*, ($\Delta t_{ji}^{spk}$), with *τ*_*f*_ = 5 *ms* and *τ*_*s*_ = 250 *ms* or *τ*_*s*_ = 30 *ms* for excitatory synaptic currents and inhibitory synaptic currents, respectively.

The ionic currents are *I*_*Na*_, *I*_*K*_ and $I_{K_{s}}$, representing sodium (Na), potassium (K), and muscarinic slow potassium (K_*s*_), respectively. More specifically: $I_{Na}=g_{Na}m_{\infty }^{3}h(V_{i}-V_{Na})$, with $m_{\infty }={\left(1+\exp\left(\frac{-V_{i}-30}{9.5}\right)\right)}^{-1}$ being the activation of the channel and where *h*, the inactivation, is given by the solution to $\frac{d}{dt}h=(h_{\infty }-h)/\tau_{h}$, with $h_{\infty }={\left(1+\exp\left(\frac{V_{i}+53}{7}\right)\right)}^{-1}$ and $\tau_{h}=0.37+2.78{\left(1+\exp\left(\frac{V_{i}+40.5}{6}\right)\right)}^{-1}$; *I*_*K*_ = *g*_*K*_*n*^4^(*V*_*i*_−*V*_*K*_) with $\frac{d}{dt}n=(n_{\infty }-n)/\tau_{n}$ where $n_{\infty }={\left(1+\exp\left(\frac{-V_{i}-30}{10}\right)\right)}^{-1}$ and $\tau_{n}=0.37+1.85{\left(1+\exp\left(\frac{V_{i}+27}{15}\right)\right)}^{-1}$; and $I_{K_{s}}=g_{Ks}s(V_{i}-V_{K})$ with $\frac{d}{dt}s=(s_{\infty }-s)/75$ where $s_{\infty }={\left(1+\exp\left(\frac{-V_{i}-39}{5}\right)\right)}^{-1}$, with the time constants being in *ms* and voltages in *mV*. The reversal potentials are *V*_*Na*_ = 55 *mV* and *V*_*K*_ = −90 *mV*, and the maximal conductances are $g_{Na}=24\frac{mS}{{cm}^{2}}$, $g_{K}=3.0\frac{mS}{{cm}^{2}}$.

The slow potassium conductance

$$g_{K_{s}}=\left\{ \begin{array}{c} 0\frac{mS}{{cm}^{2}}\;if\;ACh\;is\;high\\ 1.5\frac{mS}{{cm}^{2}}\;if\;ACh\;is\;low \end{array}\right.$$

controls the level of ACh, e.g. during wakefulness (high ACh) or NREM sleep (low ACh). The values thus control the excitability type, where low *g*_*Ks*_ (high ACh) yields Type 1 excitability and high *g*_*Ks*_ (low ACh) yields Type 2 excitability. Type 1 excitability is characterized by arbitrarily low firing frequencies, high frequency gain as a function of constant input, and a constant advance in the phase response curve whereas Type 2 has a threshold in firing frequency onset, a shallow frequency gain function, and a biphasic phase response curve. See Stiefel, Gutkin, and Sejnowski [33].

The *I*_*noise*_ is defined as a short, 1ms step current of an amplitude large enough to elicit single spike on a neuron. The timing of the step current is drawn from a Gaussian distribution so that noise events are applied with a given mean frequency (as noted on Fig 7).

Each simulation was completed using the RK4 integration method with a step size of *h* = 0.05*ms*.

### Network properties

The network used in these studies consists of *N* = 1000 neurons, with *N*_*e*_ = 800 excitatory neurons and *N*_*i*_ = 200 inhibitory neurons. Connections form a random network with different levels of connectivity dependent on the pairwise pre- and post-synaptic neuron identity: Inhibitory neurons project to 50% of the inhibitory neurons and 30% to the excitatory neurons whereas excitatory neurons project to just 6% of both the inhibitory and excitatory neurons, with self-connections being forbidden in all cases. The initial synaptic weights are *g*_*I*−*I*_ = 0.0013 *mS*/*cm*^2^, *g*_*I*−*E*_ = 0.0005 *mS*/*cm*^2^, $g_{E-I}=0.00046\frac{mS}{cm2}$, and *g*_*E*−*E*_ = 0.00003 *mS*/*cm*^2^ in Figs 1 and 2, but with *g*_*E*−*E*_ = 0.00001 *mS*/*cm*^2^ in Figs 4–7. For initial engram formation, reciprocal connections among a random subset of 200 excitatory neurons increase their *g*_*E*−*E*_ conductances, constituting the strength of the memory. The effect of the degree of increase in this synaptic strength, and as a function of number of cells affected by this strengthening is investigated in Fig 2 and elsewhere is kept at 10x.

Separately, to investigate oscillatory pattern formation in a network with no reciprocal excitatory-to-excitatory connectivity (Figs 1G–1I and 2C) we simulated initial memory formation via changes to the mean external current, I_*ext*_, each neuron receives, i.e. the external current was modified from *I*_*ext*_ ∈ [1.05, 1.10]. to *I*_*ext*_ ∈ [1.33, 1.40] with input noise of 0.5 Hz.

### Implementing STDP in the network

Neural correlates of memory are thought to emerge due to the strengthening and weakening of synaptic strengths in an activity-based manner. Here, we use a symmetric learning rule, implemented via spike timing-dependent plasticity (STDP), that uniformly increases or decreases synaptic weights based on the time-ordering of pre- and postsynaptic pair firings, only in excitatory-to-excitatory connections. If a presynaptic neuron fires before its postsynaptic partner, the conductance increases by an amount $Exp\left(-\frac{t_{pre}^{spk}-t_{post}^{spk}}{10}\right)$. Similarly, a weakening of synaptic strength occurs by an amount $\rho \;Exp\left(-\frac{t_{post}^{spk}-t_{pre}^{spk}}{10}\right)$ when a postsynaptic neuron fires before its presynaptic partner. In both cases, if the time difference between spike pairs is too great, the change in synaptic strength will approach zero. On the other hand, highly coincident spike pairs will have a maximal change given by the learning rate *ρ* = 10^−3^. It should be noted that while the synaptic weight is prohibited from becoming negative, there is no upper-bound set on the synaptic strength, though previous work has shown saturation of synaptic weights given sufficient time [14].

While simulations presented in the main manuscript (Fig 5) use standard asymmetric STDP rules described above, S2 and S3 Figs use STDP rules symmetrical around the lead/lag of the spike of the presynaptic neuron, with synaptic strength changes being only positive or negative, respectively.

### Statistical analysis

#### Analysis of functional network structures through AMD and FuNS

Average Minimal Distance (AMD) [63] was applied to network firing data to determine functional connectivity. AMD calculates the mean value of the smallest temporal difference between all spikes in one neuron and all spikes in another neuron. Analytical calculations of the expected mean and standard deviation of minimal distance is then used to rapidly determine the significance of pairwise minimal distance [25]. Specifically, the first and second raw moments of minimal distance for each node are calculated: $\mu_{1}^{L}=\frac{L}{4}$ and $\mu_{2}^{L}=\frac{L^{2}}{12}$, where L is the temporal length of the interspike interval and we have assumed that (looking both forward and backward in time) the maximum temporal distance between spikes is $\frac{L}{2}$. Over the entire recording interval T, the probability of observing an inter-spike interval of length L is simply $p(L)=\frac{L}{T}$. Then, the first and second moments of minimal distance considering the full recording interval are given as $\mu_{1}=\frac{1}{4T}\sum_{L}L^{2}$ and $\mu_{2}=\frac{1}{12T}\sum_{L}L^{3}$. Finally, the calculated statistical moments give rise to the expected mean and standard deviation, *μ* = *μ*_1_ and $\sigma =\sqrt{\mu_{2}-\mu_{1}^{2}}$, which are used to determine the Z-score significance of pairwise connectivity: $Z=\frac{{AMD}_{ij}-\mu_{i}}{\sigma_{i}}$. Values of *Z*_*ij*_≥2 represent significant functional connections between node pairs.

Functional Network Stability (FuNS) tracks global changes in network functional connectivity by quantifying similarities in AMD matrices over a recording interval. The procedure is as follows: first, a recording interval is split into n partitions of equal temporal length. Each partition is subjected to AMD functional connectivity analysis, resulting in n functional connectivity matrices, Z.

Similarity between time-adjacent functional networks is determined using the normalized dot product after matrix vectorization. FuNS is then determined by taking the mean of these cosine similarities over the recording interval: $FuNS=\frac{1}{n-1}\sum_{t=1}^{n-1}\frac{<Z_{t}|Z_{t+1}>}{\left|\left|Z_{t}\right||||Z_{t+1}|\right|}$. Thus, FuNS yield insight into how functional connectivity changes over time.

#### Spectral analysis, spike-field coherence, and phase relationships

Histograms of neuronal firing per unit time were used to calculate the network characteristic frequency, spike-field coherence, and phase relationships of individual neurons to the network signal. First, spike timings were converted into binary spike vectors and then summed to give a network spike vector. Then, the spike vector was convolved with a Gaussian distribution with zero mean and a standard deviation of ~2 ms, giving a continuous network signal. The spectral power was measured by taking the Fourier transform of the excitatory, non-engram signal (i.e. no neurons with artificially strengthened connections were used; Fig 1). Then, the change in spectral power (e.g., in Fig 2) was calculated by integrating the frequency-domain signals and taking the relevant percent difference.

Finally, phase relationships of excitatory neuron firing compared to inhibitory local field potentials was calculated (please see Fig 4A). Peaks in the inhibitory signal acted as the start and end of a given phase and excitatory spike times were used as place-markers of phase between an individual neuron and the signal. The phase of spike occurrence was normalized to give values between 0 and 1.

#### Burst detection

We analyzed bursts of activity for firing asymmetry between active neurons. First, recordings of a given interval of length L were segmented into smaller windows of length x (25 ms in CFC, 50 ms in CNO/DMSO, and 100ms in model simulations; with times chosen to maximize number of pairwise co-activations occurring) with windows overlapping by 12.5 ms to increase the sampling of the interval L and to reduce effects of windows onset. Then, the total number of **active neurons**, in each window is determined and used to define a burst-detection threshold: a burst occurs if the activity in a window is significantly greater than the mean background activity, averaged over all intervals of a given vigilance state. Specifically, if a window ***w***_***i***_ has a corresponding number of active neurons ***k***_***i***_, then the set of windows representing bursts over all intervals is given as ***B*** = {***w***_***i***_|***k***_***i***_≥***μ***_***k***_+**2*σ***_***k***_}, where ***μ***_***k***_ and ***σ***_***k***_ are the mean and standard deviation across all ***w***.

#### Firing asymmetry calculation

Next, the pairwise firing asymmetry A is calculated across all detected bursts, where A is an *N*×*N* matrix with entries ***A***_*m*,*n*_>0 if the spikes of neuron m occur before the spikes of neuron n on average, and ***A***_*m*,*n*_<0 in the opposite case. The exact value of an entry ***A***_*m*,*n*_ is given as the normalized sum of fractional differences between the number of spikes of neuron *n* occurring after and before each spike of neuron *m*, across all detected bursts B:

$$A_{m,n}=(\frac{1}{B_{m,n}})\sum \sum \frac{\left(T_{n>m}-T_{n<m}\right)}{\left(T_{n>m}+T_{n<m}\right)},$$

Where *T*_*n*>*m*_ represents the number of spikes of neuron *n* occurring after a given spike of neuron *m*, the inner sum is over the number of spikes of neuron m within a given burst, the outer sum is over all bursts, and the normalization factor, ***B***_***m*,*n***_, is the total number of bursts where neurons n and m are coactive.

#### Relating firing asymmetry to phase coding of activation frequency

The rows and columns of A are sorted by neuronal firing rate measured within a given vigilance state (i.e. wake or NREM). After sorting, firing asymmetry of slow firing rate neurons compared to high firing rate neurons will (a) compose the lower triangular matrix of *A* and (b) will be more positive than the upper triangular matrix of *A* if faster firing neurons lead slower firing neurons. We thus compared each pairwise entry of *A*_*m*,*n*_ in the lower triangular matrix with its reciprocal *A*_*n*,*m*_ in the upper triangular,

$${\tilde{A}}_{m,n}=A_{m,n}-A_{n,m}.$$

If, then the faster firing neuron leads the slower firing neuron on average.

We next determined the significance of each ${\tilde{A}}_{m,n}$ by randomizing the timing of each neuron’s spikes within each burst, 100 times (i.e. bootstrapping). The value of significance is then given by the Z-score,

$$Z({\tilde{A}}_{m,n})=\frac{{\tilde{A}}_{m,n}-{\mu ({\tilde{A}}_{m,n})}_{randomized}}{{\sigma ({\tilde{A}}_{m,n})}_{randomized}},$$

Where ${\mu ({\tilde{A}}_{m,n})}_{randomized}$ and ${\sigma ({\tilde{A}}_{m,n})}_{randomized}$ are the mean and standard deviation of the randomized distributions and with $Z({\tilde{A}}_{m,n})\geq 2$ indicating that neuron m leads neuron n in a significant way (95% confidence interval). In all, we obtain a distribution with $\frac{1}{2}N(N-1)$ elements, where N is number of detected neurons.

Finally, we compare the changes distributions of $Z({\tilde{A}}_{m,n})$ for the investigated cases (Figs 4 and 5). For this comparison we take the top 20% of the pairs that fire within the same bursts (as opposed to cells that maybe not active within the same bursts). A positive difference at $Z({\tilde{A}}_{m,n})\geq 2$ indicates an increase in fast firing neurons leading slow firing neurons within a burst of activity.

Alternatively, instead of calculating the individual pairwise differences in asymmetry, ${\tilde{A}}_{m,n}$, a global assessment of asymmetry is achieved by first averaging all the individual asymmetry values below the *m = n* diagonal (i.e., 〈*A*_*m*,*n*_〉, where *m*<*n*) and subtracting the average value from above the *m = n* diagonal (i.e., 〈*A*_*m*,*n*_〉, where *m*>*n*). Bootstrapping of spike times within bursts can then be used to determine significance of this difference, with positive values greater than or equal to a value of 2 indicating a significant global increase in fast firing neurons leading slow firing neurons within the network (Fig 4F).

#### Determining significance of firing asymmetry distributions

The firing asymmetry Z-score distributions were tested against asymmetries across the same distribution but calculated using randomized spike times. For each mouse, once bursting architecture is determined (i.e. number of bursts given interval), asymmetry was calculated using randomized spike timings. Asymmetries were then used to determine significance distributions as outline above and this process was repeated and then averaged over 25 runs. The averaged Z-score distribution is then compared to the standard, real asymmetry Z-score distribution to determine significance, using a two-sided t-test.

## Supporting information

S1 Fig The peak frequency of slow oscillations during NREM sleep like state, can be regulated via strength of inhibitory connectivity to excitatory cells (A) as well as time constants regulating decay of inhibitory postsynaptic currents (B). The specific frequency of slow oscillations during NREM sleep like state does not affect the observed dynamic and structural network reorganization.
(TIF)

S2 Fig Effects of all-potentiating STDP. **Left:** change of spiking frequencies as a function of the initial spiking frequency of the cell. **Right:** relationship between change of neuronal input (green) and output (black) and spiking frequency change.
(TIF)

S3 Fig Effects of all-depressing STDP. **Left:** Change of spiking frequencies as a function of the initial spiking frequency of the cell. **Right:** Relationship between change of neuronal input (green) and output (black) and spiking frequency change.
(TIF)
