# Supplementary figures and images for: Acetylcholine-gated current translates wake neuronal firing rate information into a spike timing-based code in Non-REM sleep, stabilizing neural network dynamics during memory consolidation
Source: PLoS Comput Biol. 2021 Sep 20;17(9):e1009424. doi: 10.1371/journal.pcbi.1009424 (PMC8483332; doi:10.1371/journal.pcbi.1009424)

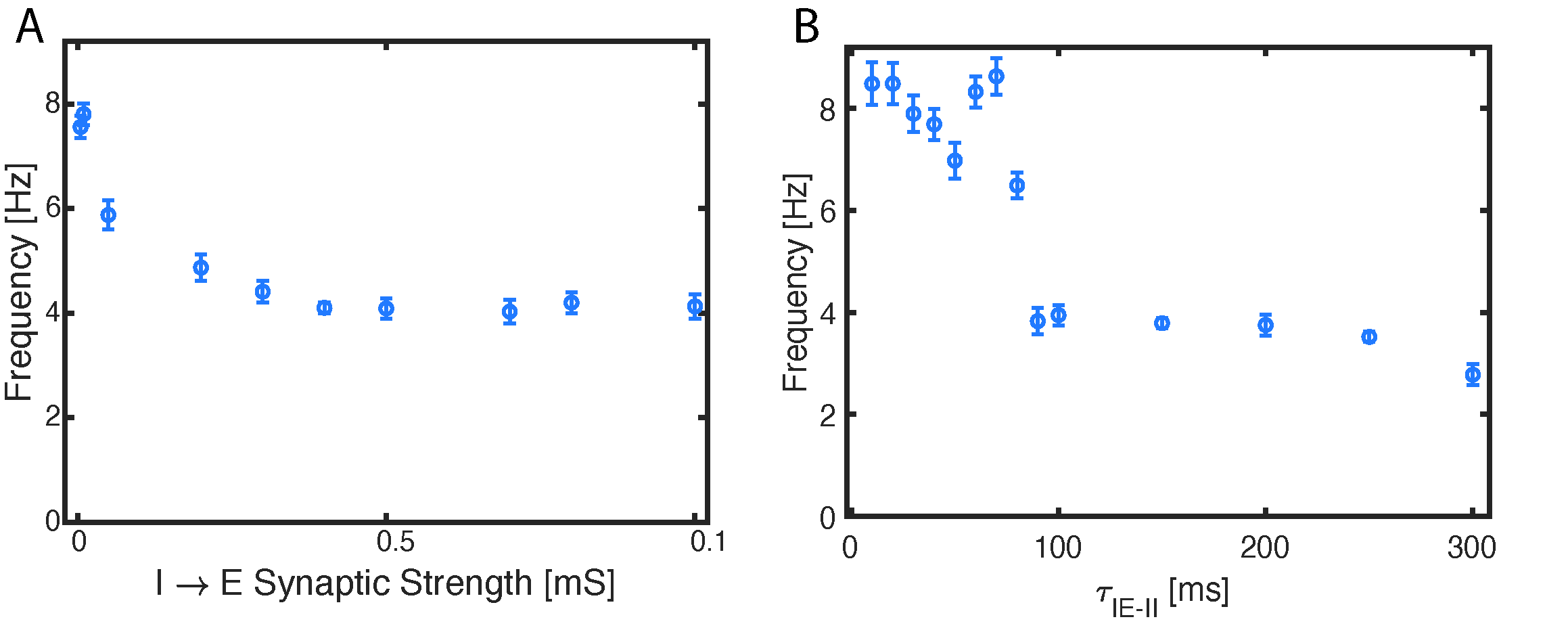

Supplement: S1 Fig — The peak frequency of slow oscillations during NREM sleep like state, can be regulated via strength of inhibitory connectivity to excitatory cells (A) as well as time constants regulating decay of inhibitory postsynaptic currents (B). The specific frequency of slow oscillations during NREM sleep like state does not affect the observed dynamic and structural network reorganization. (TIF) [file pcbi.1009424.s001.tif]

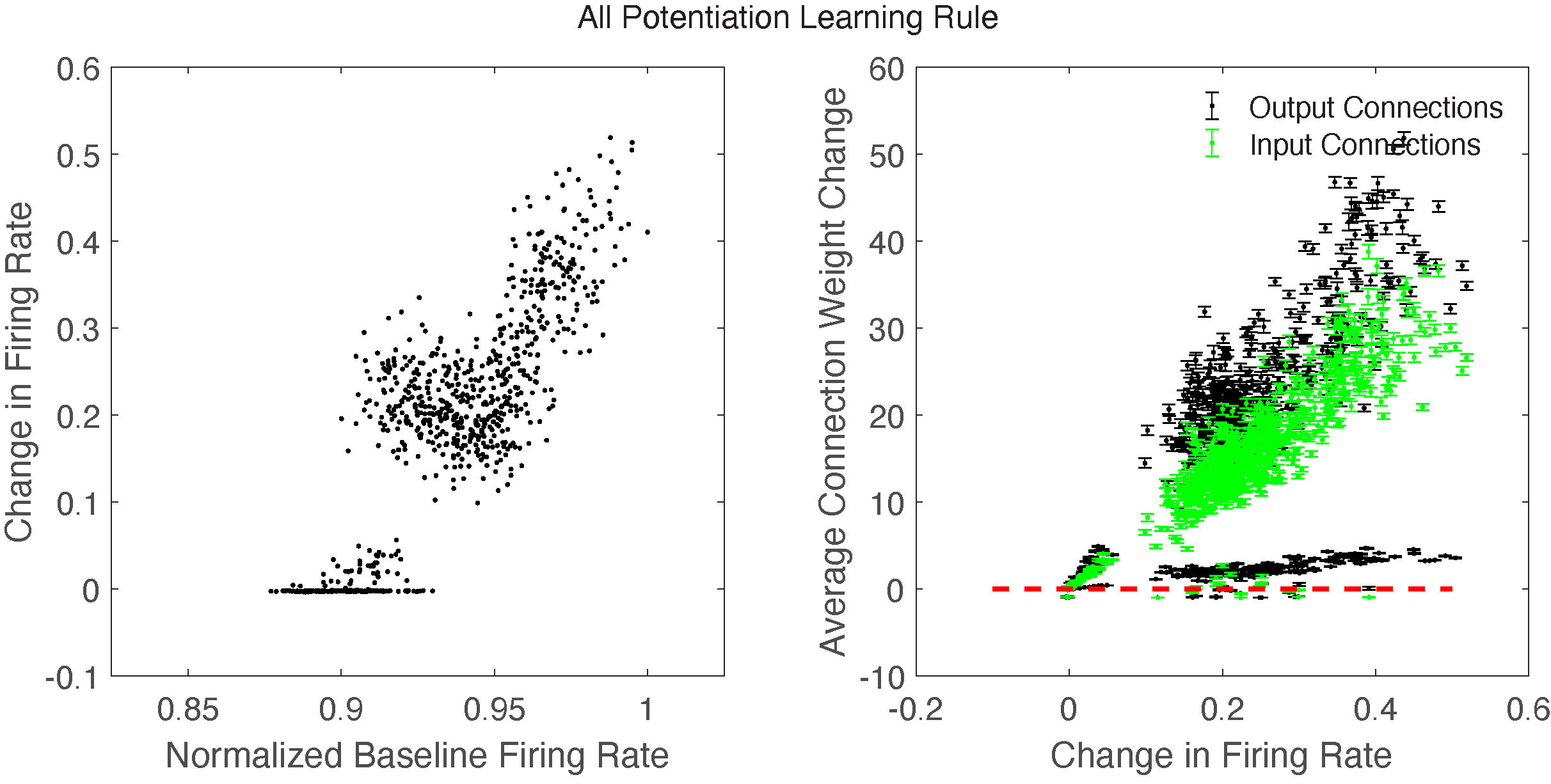

Supplement: S2 Fig — Left: change of spiking frequencies as a function of the initial spiking frequency of the cell. Right: relationship between change of neuronal input (green) and output (black) and spiking frequency change. (TIF) [file pcbi.1009424.s002.tif]

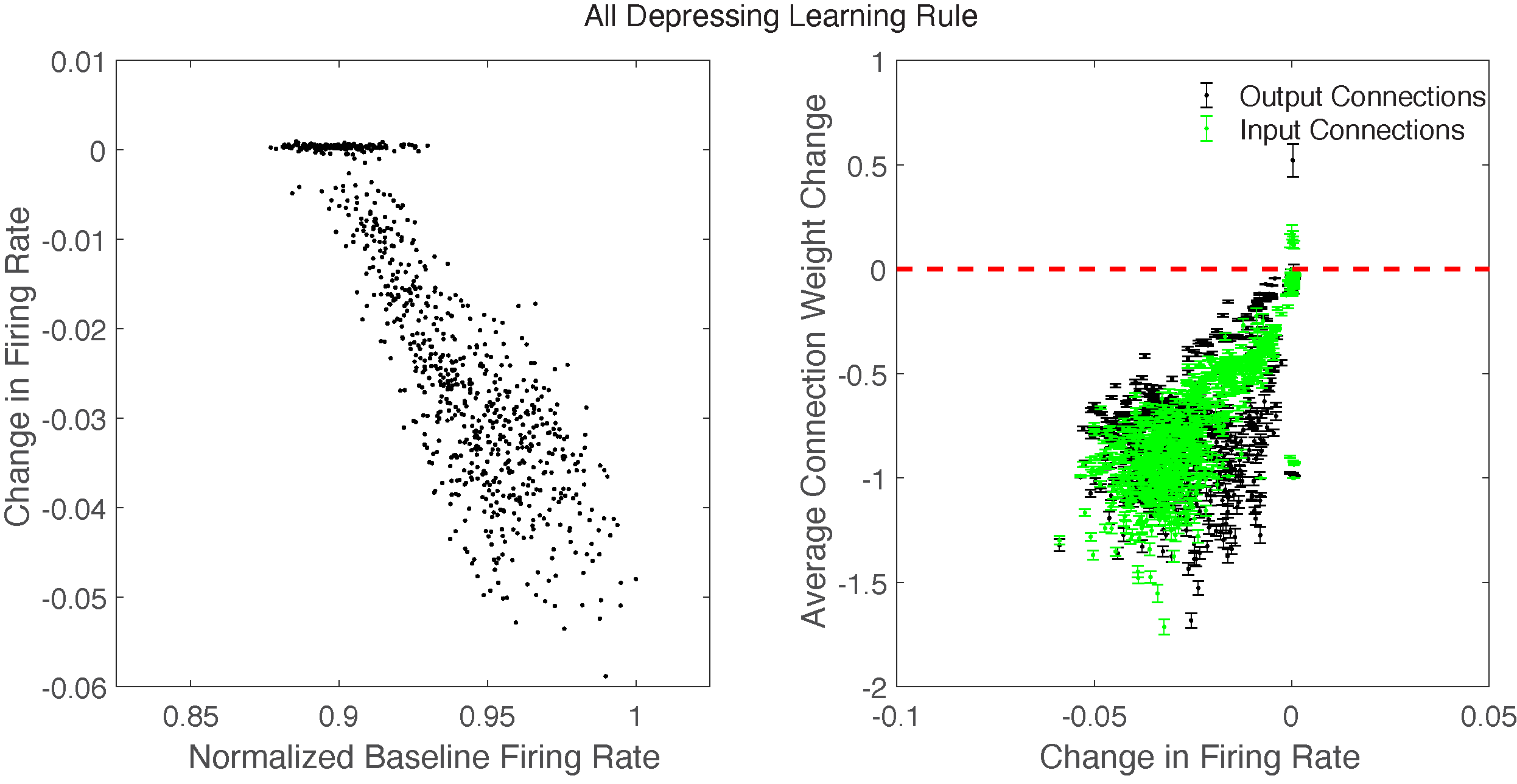

Supplement: S3 Fig — Left: Change of spiking frequencies as a function of the initial spiking frequency of the cell. Right: Relationship between change of neuronal input (green) and output (black) and spiking frequency change. (TIF) [file pcbi.1009424.s003.tif]
